# Osteoporosis Improved by Romosozumab Therapy in a Patient With Type I Osteogenesis Imperfecta

**DOI:** 10.1016/j.aace.2023.10.002

**Published:** 2023-10-27

**Authors:** Antara Dattagupta, Steven Petak

**Affiliations:** 1 Division of Endocrinology, Diabetes, and Metabolism, Department of Medicine, Houston Methodist Hospital, Houston, Texas; 2 Washington University School of Medicine, Department of Medicine, St. Louis, Missouri

**Keywords:** osteogenesis imperfecta, romosozumab, bone mineral density

## Abstract

**Background/Objective:**

Osteogenesis imperfecta (OI) is a genetic disorder that affects type 1 collagen synthesis causing increased bone fragility, low bone mass, and skeletal deformity. Bisphosphonates are recommended for treatment of OI patients; however, the efficacy of sclerostin inhibitors such as romosozumab has not been determined in OI patients with osteoporosis.

**Case Report:**

A 52-year-old G2P2 clinically diagnosed with OI, with a history of multiple fractures beginning in childhood presented with low bone mass. On physical examination, blue sclera was observed. She was previously treated with alendronate therapy from April 2014 to June 2015 without significant improvement in bone mineral density (BMD). After the onset of menopause, she began romosozumab 210 mg subcutaneous therapy once a month for 12 months. Repeat dual-energy X-ray absorptiometry showed an increase of 10.3% in BMD of the spine and a 5.4% increase in BMD of the right hip. The trabecular bone score increased by 5.2%.

**Discussion:**

Current literature is limited regarding the use of sclerostin inhibitors in OI patients. Our patient’s improvement in BMD of the spine and right hip after romosozumab therapy was significant at a 95% confidence level, compared to treatment initiation. Her trabecular bone score also improved significantly. Six months into our patient’s treatment course, a case in Japan of a male with severe osteoporotic OI and recurrent fractures showed improvement in BMD after romosozumab therapy.

**Conclusion:**

This case highlights our patient’s significant response to romosozumab and warrants further investigation of romosozumab as a potential treatment option for OI patients with osteoporosis.

**Highlights**

• Impact of romosozumab therapy in osteogenesis imperfecta patients with osteoporosis
• Suggested expansion of indications for romosozumab therapy
• Improvement in the bone mineral density by romosozumab regardless of osteogenesis imperfecta subtype and severity

**Clinical Relevance**

We describe a patient’s significant response to romosozumab with no adverse effects noted suggesting further investigation of romosozumab as a potential treatment option for osteogenesis imperfecta (OI) patients with osteoporosis. Here, we also report the second known case of significant improvement in bone mineral density using romosozumab in OI patients with osteoporosis.

## Introduction

Osteogenesis imperfecta (OI), also known as brittle bone disease, is one of the most common genetic bone disorders, with a prevalence of approximately 1 in 10 000 to 20 000 births.^1^ OI affects type 1 collagen synthesis, causing increased bone fragility, low bone mass, and skeletal deformity. The severity of OI ranges from mild to perinatal death and has been classified by the Sillence classification, which describes the classic features and clinical severity of OI based on its type. Bisphosphonates are recommended for treatment of OI, because they increase bone mineral density (BMD) and decrease fracture risk with a minimal adverse drug profile.^2^

A study in 2011^3^ investigating fractures and bone mass in OI showed that using the WHO criteria on total body BMD, about 30% of the OI population had osteopenia and 10% were osteoporotic. Females with OI may present with worsening osteoporosis during gestation, lactation, and after menopause.^4^ Osteoporosis is a multifactorial disease in which bone strength is compromised, thus predisposing patients to an increased fracture risk.^5^ Osteoporosis results from osteoclastic bone resorption that is not compensated by osteoblastic bone formation. Historically, osteoporosis affects women more than men. Common causes include estrogen deficiency, reduced dietary intake, and vitamin D deficiency. Treatment options include lifestyle changes and/or medications. Lifestyle changes involve exercise, smoking and alcohol cessation, and appropriate dietary intake. Medications can be classified according to their mechanism of action: antiresorptives (bisphosphonates and denosumab), anabolics (teriparatide and abaloparatide), and dual-action agents (romosozumab).

Romosozumab is a dual-action agent that exhibits both anabolic and antiresorptive properties by increasing bone formation and decreasing bone resorption.^6^ Specifically, the Wnt signaling pathway serves to remodel bone and increase skeletal development.^7^ This pathway is inhibited by sclerostin. Romosozumab is a monoclonal antibody that binds to sclerostin, thus preventing sclerostin from inhibiting the Wnt pathway and overall promoting bone formation and remodeling.^7^ This therapy was approved for high-risk postmenopausal females with osteoporosis as well as patients who have experienced treatment failures with other osteoporosis therapies.^6^ A randomized controlled trial in 2016 showed that romosozumab was associated with a lower risk of vertebral fracture at 12 months in postmenopausal women with osteoporosis than placebo.^8^

Although bisphosphonates are the recommended treatment for OI patients, the efficacy of romosozumab has not been established in OI patients with osteoporosis. Herein, we present a case of osteoporosis in a patient with clinically diagnosed type 1 OI who improved significantly on romosozumab therapy.

## Case Report

A 52-year-old G2P2 clinically diagnosed with OI is being followed by the endocrinology department for low bone mass. Her medical history is significant for multiple reported fractures in childhood, a fibula fracture in 1998, and a rib fracture due to coughing in 2013. She has a history of multiple knee and foot surgeries, the most recent of which was a tendon reattachment in 2018. She is a nonsmoker with minimal alcohol use. She denies any previous glucocorticoid use or eating disorders. She endorses regular menstrual cycles before recent-onset menopause in 2021 at the age of 51 years. She takes Vitamin D3 2000 IU daily. She previously underwent alendronate therapy from April 2014 to June 2015 without significant improvement in BMD. Her family history is significant because her mother had a hip fracture and osteoporosis around the age of 60 years. On physical examination, blue sclera was observed (Fig.). She was 5 feet 2 inches tall and weighed 109 pounds upon presentation. All other physical examination findings are unremarkable.

**Fig. 1:**
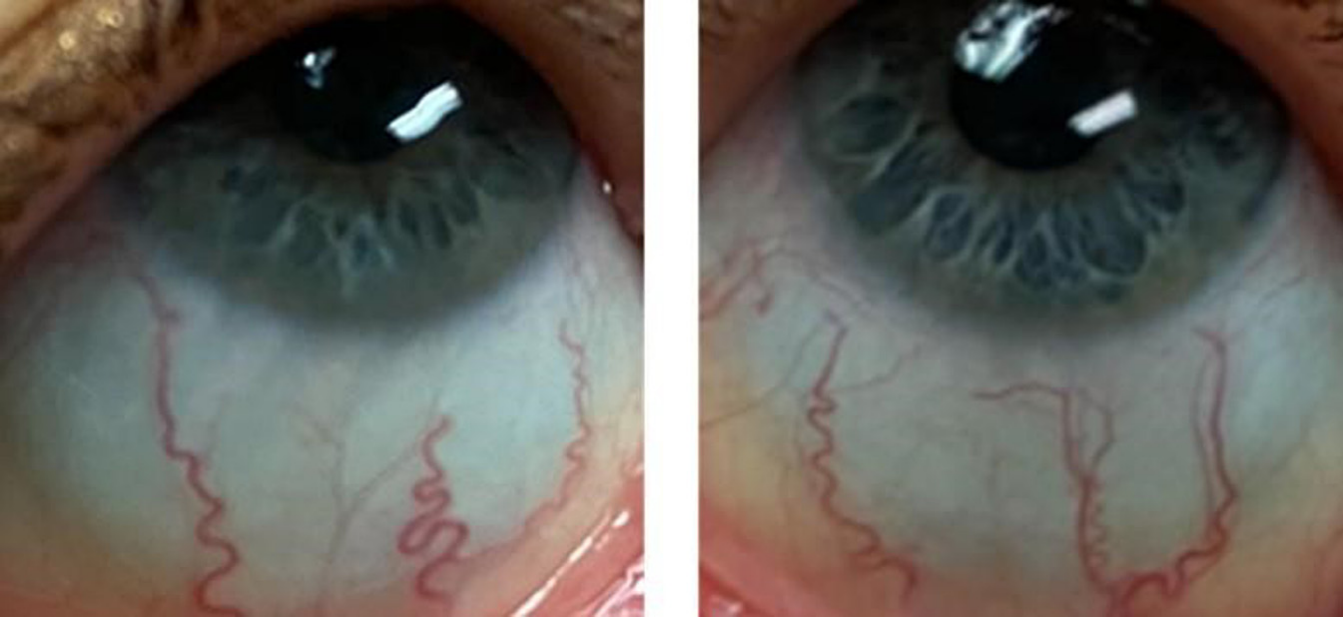
Clinical finding of osteogenesis imperfecta: presence of blue sclera.

Our patient had a stable decline of bone mass throughout the years, which was followed by yearly dual-energy X-ray absorptiometry (DXA) scans. However, after her menopause in 2021, her DXA scan showed increased worsening BMD. Her BMD change compared with her previous DXA scan in 2020 indicated an 11.3% decline in her L1 to L4 spine and a 6% decline of her total left hip both statistically significant to the 95% confidence level, where the least significant change (LSC) for AP spine is 0.022 g/cm^2^ and LSC for total hip is 0.027 g/cm^2^ (Table 1). LSC is the minimum amount of bone mineral density change considered to be statistically significant.

**Table 1:** Bone Mineral Density (BMD) Change Compared With Previous Year on DXA

Region	Exam year	BMD change vs previous
AP spine (L1-L4)	2022 (after romosozumab)	+10.3%∗
	2021 (before romosozumab)	-11.3%∗
	2020	1.8%
Total Hip (Right)	2022 (after romosozumab)	5.4%∗
	2021 (before romosozumab)	-2.7%
	2020	4.1%
Total Hip (Left)	2022 (after romosozumab)	4.5%
	2021 (before romosozumab)	-6.0%∗
	2020	-1.1%

∗ Denotes significance at 95% confidence level where least significant chance (LSC) for AP spine = 0.022 g/cm^2^ and LSC for total hip = 0.027 g/cm^2^.

The patient’s minimal response to bisphosphonates and the recent onset of menopause led to the decision to start dual-action therapy. She started on romosozumab 210 mg subcutaneous therapy once a month for 12 months, followed by a repeat DXA after completion of the treatment course (Table 2). She tolerated the therapy without any adverse effects. Her calcium, alkaline phosphatase, and bone-specific alkaline phosphatase laboratory values before and after romosozumab therapy were within normal limits. According to her DXA results, she had an increase of 10.3% in BMD of the spine and a 5.4% increase in BMD of the right hip, both significant at a 95% confidence level compared with treatment initiation, where the LSC for AP spine is 0.022 g/cm^2^ and the LSC for total hip is 0.027 g/cm^2^ (Table 1). Trabecular bone score, which measures the quality of the bone, increased to 1.306, improving by 5.2%, where LSC is 4.2%.

**Table 2:** DXA comparing pre- vs postromosozumab course

Region	06/2021 BMD (g/cm^2^)	06/2021 T-Score (Before romosozumab)	07/2022 BMD (g/cm^2^)	07/2022 T-Score (After romosozumab)
AP spine (L1-L4)	0.648	-3.6	0.715	-3.0
Femoral neck (left)	0.435	-3.7	0.440	-3.7
Total hip (left)	0.530	-3.4	0.554	-3.2
Femoral neck (right)	0.456	-3.5	0.489	-3.2
Total hip (right)	0.559	-3.1	0.589	-2.9
Total hip mean	0.545	-3.3	0.572	-3.1

After successfully completing the romosozumab course without any reported adverse effects, our patient began annual zoledronate therapy for 2 to 3 years to maintain benefit. To our knowledge, she has not had another fracture after her treatment course. Her BMD will be closely monitored with yearly DXA, and the possibility of a second round of romosozumab therapy in the future will be explored if indicated.

## Discussion

Our patient did not have genetic confirmation of OI at this time due to financial reasons. However, using the Sillence classification of OI, our patient was clinically diagnosed with mild nondeforming type 1 OI with the typical features of normal height, blue sclera, and absence of dentinogenesis imperfecta.^9^ With her stable decline in bone mass and absence of any recent fractures, our patient’s BMD was being monitored closely with yearly DXA scans without any osteoporosis medications since her bisphosphonate trial from April 2014 to June 2015. However, after the onset of menopause, our patient lost the protective effect of estrogen and had increased worsening of her BMD as observed with her DXA scan, indicating that therapy was necessary.

Initiating therapy for patients with OI is determined on a case-by-case basis. The goals of therapy are to decrease fracture incidence, decrease pain, increase mobility, improve bone metabolic markers, and increase patient independence. Bisphosphonates, which are currently recommended for OI, inhibit bone resorption, thus decreasing the number and activity of osteoclasts through apoptosis, which reduces bone turnover.^10^ Other pharmacologic treatments, such as teriparatide, are being investigated in patients with OI. Teriparatide, an anabolic, showed improvement in increasing BMD for adult patients with mild OI such as our patient but was not effective for patients with moderate or severe OI.^11^ Several recent studies using teriparatide have showed positive effects on bone density in mostly type I OI patients; however, none of these studies determined the effect of teriparatide on fracture risk reduction.^12^

Romosozumab is a sclerostin inhibitor, which stimulates bone formation by osteoblasts while also inhibiting bone resorption by osteoclasts thus exhibiting both anabolic and antiresorptive properties.^13^ Current literature is limited regarding the use of sclerostin inhibitors in OI patients. Therefore, our patient’s worsening osteoporosis, before the bisphosphonate trial, and postmenopausal status suggested that she would be a high-risk fracture candidate and could benefit from romosozumab therapy.

Six months into our patient’s treatment course, a case in Japan was published of a 64-year-old male with severe osteoporotic OI and recurrent fractures who also showed significant improvement in BMD after romosozumab therapy.^14^ His lumbar and total hip BMD increased by 22% and 136.4%, respectively. This case supports our findings of significant improvement in BMD in patients with OI after romosozumab therapy and warrants future research in expanding the indication of romosozumab therapy. Of note, this patient had a severe type of OI whereas our patient had mild type of OI, and both showed significant improvement after romosozumab therapy. This is a different outcome than teriparatide’s effect on patients with varying severity of OI as mentioned previously. In addition, both cases emphasize the need to explore the benefits of romosozumab therapy in OI patients compared with the current recommendation of bisphosphonates.

## Conclusion

Our patient’s significant response to romosozumab, in terms of BMD, with no adverse effects noted suggests that further investigation of romosozumab as a potential treatment option for OI patients with osteoporosis is warranted. Here, we also report the second known case of significant improvement in BMD using romosozumab in an OI patient with osteoporosis.

## Disclosure

Dr Petak is a speaker for Alexion and AMGEN. The other author has no conflicts of interest to disclose.
